# De novo myeloid sarcoma mimicking gynecological tumors: a retrospective case series of eight patients

**DOI:** 10.1186/s12905-023-02278-3

**Published:** 2023-03-29

**Authors:** Yu Gu, Haoran Zheng, Shengwei Mo, Tao Guo, Lihua Chen, Junjun Yang, Yang Xiang

**Affiliations:** 1 Department of Obstetrics and Gynecology, Peking Union Medical College Hospital, National Clinical Research Center for Obstetric & Gynecologic Diseases Chinese Academy of Medical Sciences & Peking Union Medical College, Beijing, China; 2 Chinese Academy of Medical Sciences & Peking Union Medical College, Beijing, China; 3 Department of Pathology, Peking Union Medical College Hospital, Chinese Academy of Medical Sciences & Peking Union Medical College, Beijing, China

**Keywords:** Myeloid sarcoma, Gynecological tumors, Case series, Symptoms, Diagnosis

## Abstract

**Objective:**

To describe myeloid sarcoma (MS) that mimic gynecological tumors and provide guidelines for improving the diagnosis and treatment of patients.

**Methods:**

This case series study retrospectively analyzed the clinicopathological characteristics and oncological outcomes of female patients who were histologically diagnosed with MS after initially presenting with reproductive-system tumors at the Peking Union Medical College Hospital between January 2000 and March 2022.

**Results:**

There were eight cases in which MS mimicked cervical cancer, ovarian cancer, or hysteromyoma. Six patients had isolated MS, and the other two had acute myeloid leukemia (AML)-M2. The average age was 39.00 ± 14.26. They each sought advice from a gynecological oncologist at the initial visit, complaining of irregular bleeding (3/8), low abdominal pain (3/8), dysmenorrhea (1/8), or an accidentally found mass (1/8). CT/MRI exams revealed that the average tumor size reached 5.65 ± 2.35 cm, with 50% of the tumors being larger than 8 cm. The final diagnoses were confirmed by biopsy (2/8) or postoperative pathology (6/8); the most frequent positive immunohistochemical markers were Ki-67 (60–90%), MPO (100%), LCA (62.5%), CD43 (62.5%), CD117 (62.5%), CD99 (50%), vimentin (37.5%), and lysozyme (25%). MLL/AF9 gene fusions and CEBPA, JAK2, NRAS, and FLT3-TKD mutations were found in the patients. Six (75%) of the patients showed a complete response after upfront treatment using chemotherapy + surgery and experienced no recurrence during follow-up. The overall survival (OS) rate was 72.9%, and the 5-year OS rate was 72.9% (95%CI: 0.4056–1.000). The median OS was 26 months (range: 3–82).

**Conclusion:**

For patients with isolated MS, treatment by chemotherapy and surgery are radical procedure, and initial treatment using chemotherapy alone should be considered for MS with synchronous intramedullary AML. Poor response to chemotherapy, short interval to leukemia occurrence, and heavy tumor burden (> 10 cm) could indicate a poor prognosis for patients with MS.

## Background

Myeloid sarcoma (MS), also known as granulocytic sarcoma or chloroma, is more accurately termed as extramedullary acute myeloid leukemia (AML) and has an incidence rate of 1–3% in AML patients [1]. MS is thought to originate from the invasion of primitive granulocytes or immature myeloid cells into extramedullary tissues such as the genital tract, skin, or gingiva [2]. There are two main types of MS: most MS are synchronous intramedullary AML, but in rare circumstances (< 1%), isolated MS (iMS) can occur presenting as an isolated tissue mass without bone marrow involvement [3, 4].

MS manifests as a broad heterogeneous category consisting of distinct clinical scenarios with diverse sites and clinical implications; and this adds challenges to accurate diagnosis, prognostication, and treatment. Studies have reported some cases of iMS being located in the connective tissue, gastrointestinal system, bone, brain, skin, head and neck, and reproductive system [5–12]. Patients with iMS may develop AML throughout the disease course, with an average onset of 7.4 months [1].

Although very rare, gynecological oncologists may encounter patients with MS that mimics gynecological tumor, such as a pelvic, cervical, vaginal, vulvar, and even placental mass [13–16]. However, no specific treatment guidelines are available for these patients. This case series reports eight cases of MS of the female reproductive system and analyzes the clinicopathological characteristics to provide data for guiding patient diagnosis and treatment.

## Methods

### Ethics

This retrospective study was approved by the Institutional Review Board of Peking Union Medical College Hospital. All procedures in the study involving human participants were performed in accordance with the World Medical Association Declaration of Helsinki on Ethical Principles for Medical Research Involving Humans.

### Patient selection

This case series study retrospectively reviewed female patients who were histologically diagnosed with MS initially presumed as reproductive-system tumors at the Peking Union Medical College Hospital between January 2000 and March 2022. The inclusion criteria were as follows: 1) an initial visit to the gynecology department; 2) complaints about gynecological symptoms, such as irregular bleeding and pelvic mass; and 3) histological or cytological confirmation of MS. Patients without measurable reproductive-system tumors were excluded.

### Data collection

All clinical data were extracted from electronic records, including the date of the first visit, date of histological diagnosis, interval from the first symptoms to initial chemotherapy, tumor size, medical intervention, and oncological outcomes. The follow-up information was updated to March 2022 via electronic records or phone calls. Overall survival (OS) was defined as the time from the initiation of first-line therapy to the date of death or the last follow-up.

### Statistical analysis

Descriptive statistics were calculated using SPSS Statistics version 26 (IBM Corp., Armonk, NY, USA). Continuous variables are expressed as mean ± standard deviation for data with a normal distribution or as the median and range for data not normally distributed. Categorical variables are presented as numbers and percentages. The life table was used to survival analysis.

## Results

A total of ten patients were identified initially, but two patients were excluded, as no extramedullary tumors were identified in the genital tract. Eventually, eight patients were eligible for the study. The clinical characteristics of the patients are summarized in Table 1.

**Table 1:** Clinical characteristics of each patient

**Patients**	**Age**	**Main complains**	**tumor location**	**Tumor size(cm)**	**Blood test**	**Suspected disease**	**Gynecologic intervention**	**Diagnosis**^**b**^	**Subtype**^**b**^	**M**^**c**^
P1	35	left lower abdominal pain	pelvic mass	10	negative	ovarian tumor	transabdominal pelvic mass resection	MS	iMS	negative
P2	41	dysmenorrhea	uterine mass	8.8	negative	hysteromyoma	LH+BSO^a^	MS	iMS	negative
P3	29	accidentally-found cervical mass during delivery	cervical mass	4	negative	cervical cancer	cervical biopsy+LH+BSO	MS	iMS	negative
P4	63	postmenopausal bleeding	cervical and vaginal mass	5; 5	leukocytosis; thrombocytopenia	cervical cancer	cervical biopsy	MS	AML-M2	AML-M2
P5	51	right lower abdominal pain	right adnexal masses	8	Not recorded	ovarian tumor	transabdominal right ovary and omentum resection	MS	iMS	negative
P6	31	lower abdominal pain	bilateral adnexal masses	5.6; 2.7	mild anemia	ovarian tumor	laparoscopic bilateral tumor resection	MS	iMS	negative
P7	17	irregular bleeding	cervical mass	8.4	severe anemia	cervical cancer	cervical biopsy+LH+BS	MS	iMS	negative
P8	45	irregular bleeding	cervical mass	5.7	negative	cervical cancer	cervical biopsy	MS	AML-M2	negative

**Patients**	**I**^**c**^	**C**^**c**^	**M**^**c**^	**Risk**	**Chemothrapy**	**Response**^**d**^	**Recurrency**	**Outcome**
P1	negative	46，XX	negative	intermediate	DA^e^1+HD-Ara-C^e^1+HE^e^1	PD	—	death
P2	negative	46，XX	negative	intermediate	DA^e^1+HD-Ara-C^e^4	CR	NO	survive
P3	negative	46，XX	negative	intermediate	DA^e^1+HD-Ara-C^e^4	CR	NO	survive
P4	AML-M2	46，XX	MLL/AF9 fuse gene	intermediate	Hydroxycarbamide^e^1	PD	—	death
P5	negative	46，XX	negative	intermediate	IA14^e^1+HD-Ara-C^e^4	CR	NO	survive
P6	negative	46，XX	JAK2 mutation	intermediate	IA14^e^1+HD-Ara-C^e^4	CR	NO	survive
P7	negative	46，XX	negative	intermediate	IA14^e^1+HD-Ara-C^e^4	CR	NO	survive
P8	1.5% abnormal early myeloid cell	46,XX,-inv(9)(p12q13)c,+mar[10]/46,XX,inv(9)(p12q13)c[10]^e^	CEBPA, NRAS and FLT3-TKD mutation	favorable	IA14^e^1+HD-Ara-C^e^2	CR	NO	survive

*DA* Daunorubicin and Cytarabine, *IA* Idarubicin and Cytarabine

^a^*LH* laparoscopic hysterectomy, *BSO* bilateral salpingo-oophorectomy

^b^*MS* myeloid sarcoma, *iMS* isolated MS, *AML-M2* acute myeloid leukemia-M2

^c^*MICM type* Morphology, Immunology, Cytogenetics, and Molecular biology

^d^*PD* progressive disease, *CR* complete response

^e^this karyotype is clinically meaningless

**Fig. 1:**
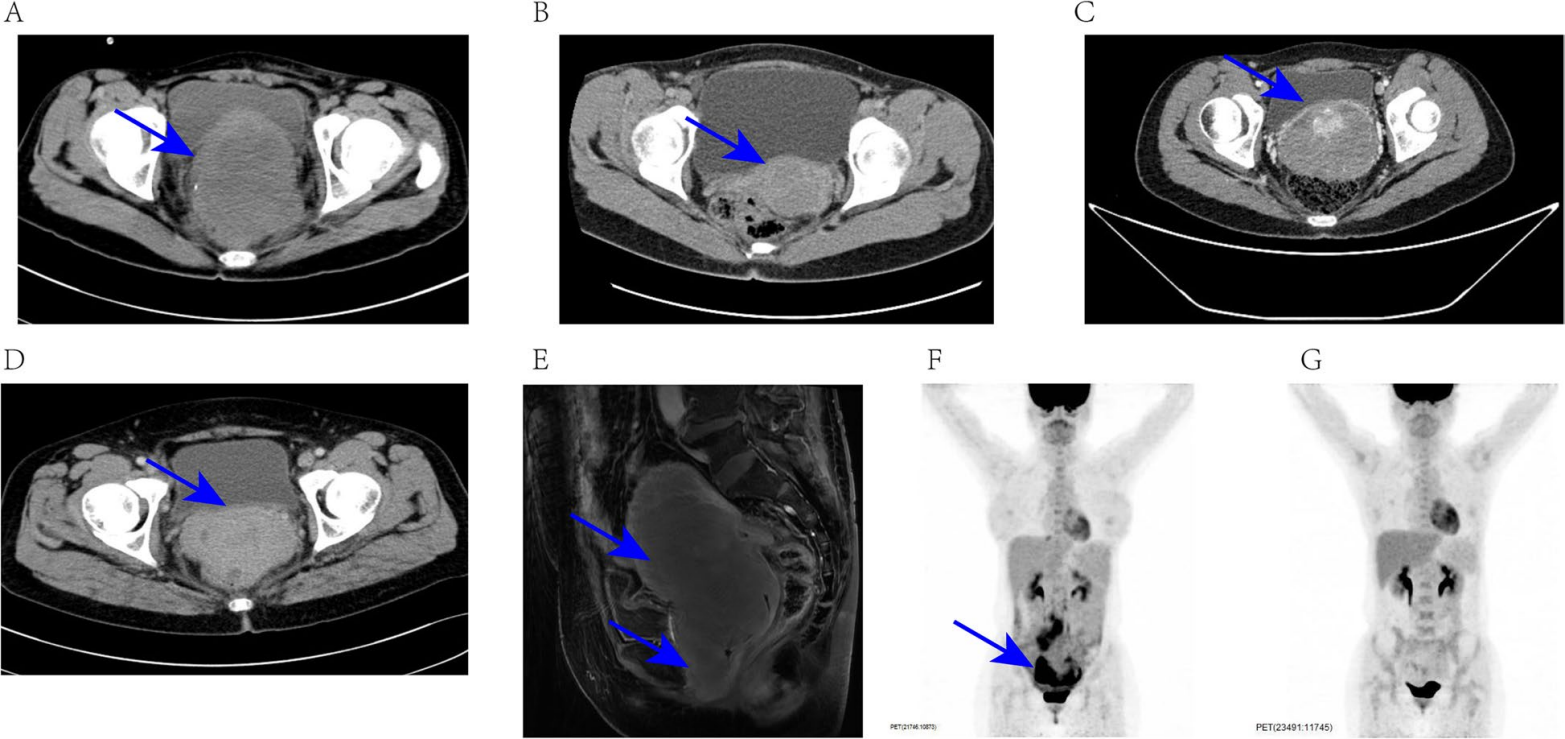
CT/MRI/PET-CT images of patients. **A** CT image of pelvic mess from patient 1(tumor size = 10 cm); **B** CT image of cervical mess from patient 3 (tumor size = 4 cm); **C** CT image of cervical mess from patient 7 (tumor size = 8.4 cm); **D** CT image of cervical mess from patient 8 (tumor size = 5.7 cm); **E** MRI image of cervical and vaginal mess from patient 4 (tumor size = 5 cm, 5 cm); **F** (before treatment) and **G** (after treatment) are PET-CT images of body from patients 6

The average age of the eight patients was 39.00 ± 14.26, and the main complaints were bleeding (3/8), pain (3/8), dysmenorrhea (1/8), and an accidentally found mass (1/8). The patients presented with measurable tumors either in the cervix/vagina suspected as cervical cancer (4/8), in the adnexa area suspected as ovarian cancer (3/8), or in the uterus suspected as hysteromyoma (1/8). The average tumor size was 5.65 ± 2.35 cm, and 50% of the tumors were larger than eight cm. Only three patients showed abnormal blood test results at the first visit. All patients had de novo MS without a history of an antecedent hematological disorder. Images of typical tumors are shown in Fig. 1A–E.

**Fig. 2:**
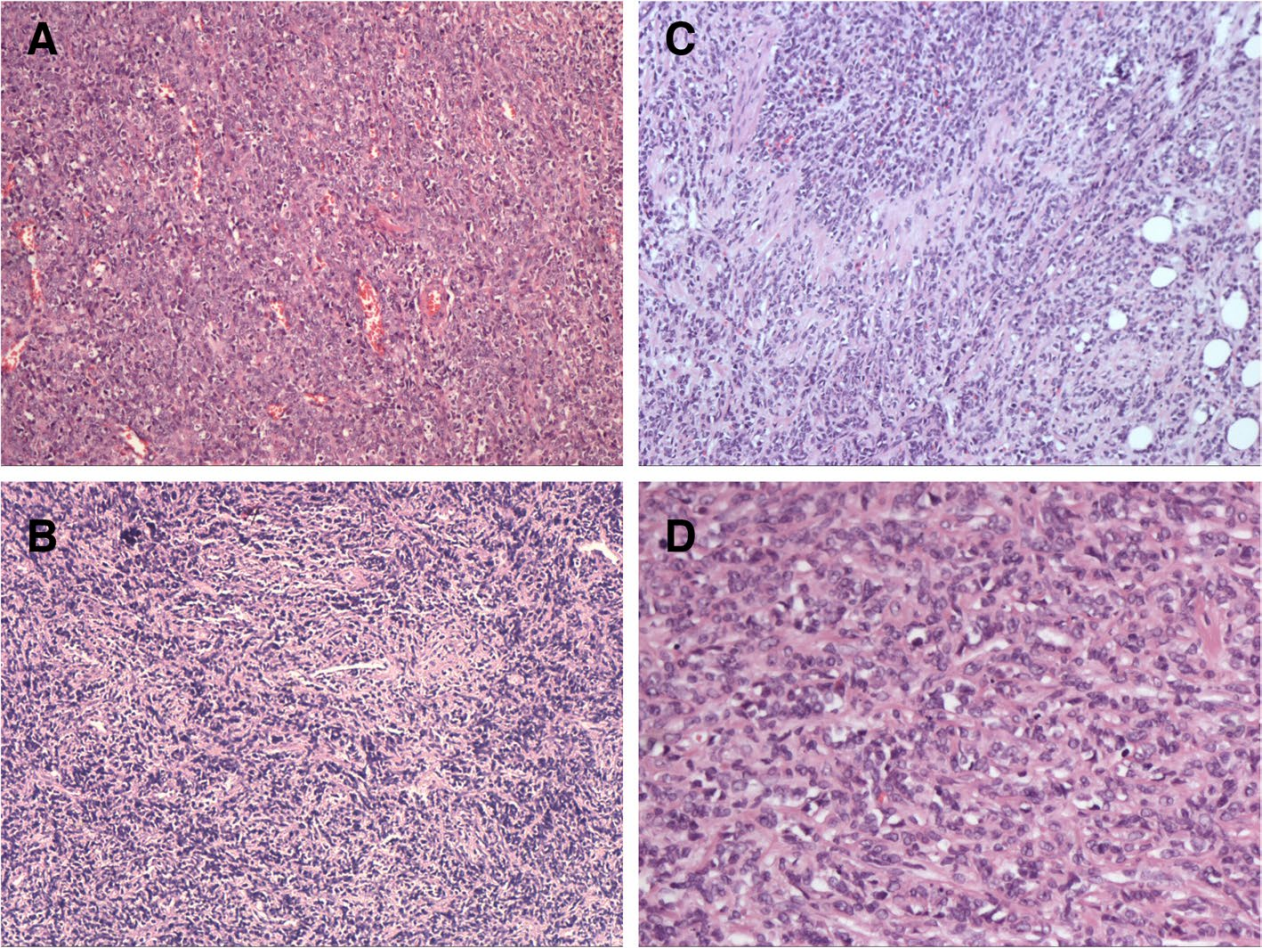
HE staining images of tumor. **A** MS in cervix from patient 3 (4 ×); **B** MS in cervix from patient 4 (4 ×); **C** MS in ovaries from patient 5 (20 ×); **D** the omentum metastasis of MS from patient 5 (40 ×)

All patients were confirmed histologically as MS, six patients by surgery and two patients by biopsy (Fig. 2). The most frequent positive immunohistochemical markers were Ki-67 (60–90%), MPO (100%), LCA (62.5%), CD43 (62.5%), CD117 (62.5%), CD99 (50%), vimentin (37.5%), and lysozyme (25%) (Table 2). Among the six patients who underwent surgery before chemotherapy, two underwent tumor resection and four underwent uterus or ovary removal. Among the subtypes of MS, six patients were confirmed to have iMS and the other two were confirmed to have AML-M2.

**Table 2:** Immunohistochemical staining results of the eight patients

	Ki-67	MPO	LCA	CD43	CD117	CD99	Vimentin	Lysozyme	Bcl-2	CD3	CD13	CD20	CD34	CD56	Syn	Bcl-6	C-MYC	Fli-1	WT-1	CYCLIND1	CD2	CD5	CD7	CD10
P1	90%	+					+			partly +				partly +		partly +				sporadic +	partly +	partly +	+	partly +
P2	60%	+			+						+		+											
P3	70%	+									+													
P4	90%	partly +	+	+	+	+				+		+												
P5	60%	+	+	+	+	+		+	+	sporadic +		sporadic +	partly +				+	+	sporadic +					
P6	90%	+	+	+	+	+	+	+	+															
P7	70%	+	+	+	partly +	+	+							+	Partly +									
P8	90%	+	+	+																				

“ + ” means the marker was positive in IHC

*Ki-67*, Ki-67 labeling index, *MPO* myeloperoxidase, *LCA* leucocyte common antigen, *Bcl* B-cell lymphoma, *Syn* synapsin, *Fli-1* Friend leukaemia integration-1, *WT-1* Wilms tumor protein-1

After a final diagnosis was obtained, each patient was transferred to the hematology department for first-line chemotherapy. The median interval between the first symptoms and initial chemotherapy was 3.5 months (range: 2–9); the median interval between the first symptoms and the initial visit to the hospital was 27 days (range: 0–178). In terms of risk stratification based on MICM classification (morphology, immunology, cytogenetics, and molecular biology), one patient had a CEBPA gene mutation and belonged to the favorable prognosis group, and the other seven patients belonged to the intermediate prognosis group; MLL/AF9 gene fusions and JAK2, NRAS, and FLT3-TKD mutations were found in these patients. Overall, six (75%) patients achieved a complete response after chemotherapy and experienced no recurrence during follow-up (Fig. 1F–G). Patients 1 and 4 showed disease progression during chemotherapy. Patient 1 underwent mass resection for iMS before chemotherapy. Patient 4 had AML-M2 and there was no chance for surgery due to rapid disease maturation. Both these patients died. At the last follow-up, the overall OS rate 72.9%, and the 5-year OS was 72.9% (95%CI: 0.4056–1.000). The median OS was 26 months (range: 3–82), and the median follow-up time was 26 months (range: 3–82).

## Discussion

This case series study identified eight cases in which MS mimicked cervical cancer, ovarian cancer, or hysteromyoma. All patient sought advice from a gynecological oncologist at their initial visit, complaining of irregular bleeding or abdominal pain. Images revealed that the average tumor size reached 5.65 cm. The final diagnoses were confirmed by biopsy (2/8) or postoperative pathology (6/8); each patient was transferred to the hematology department for radical chemotherapy. Six (75%) of the eight patients showed complete response to chemotherapy and experienced no recurrence during follow-up. The 5-year OS was 72.9%.

In total, this study reviewed patients admitted to the gynecologic department of Peking Union Medical College Hospital from 2000 to 2022 and found four patients with MS among 14,000 suspected cervical cancer cases, three among 18,000 suspected ovarian cancer cases, and one among 23,000 suspected hysteromyoma cases. Hither to, gynecological oncologists are not familiar to the diagnosis and treatment of patients with isolated MS, as well as those combined with intramedullary AML. We consider the time of pathological diagnosis as “the trigger point” when patients should be transferred to the hematology department for radical chemotherapy after gynecological interventions; the time of transfer was determined by the site of tumor. The four patients in whom the tumor presented as cervical masses promptly underwent cervical biopsy; the four cases that presented as pelvic masses and hysteromyomas were diagnosed after gynecological surgery. Currently, the diagnosis of iMS can only be confirmed with histological examination and immunohistochemistry with markers including CD34, MPO, CD117, and CD33 [1]. Previously reported positive rates for MPO (50–88%), CD34 (22–44%), CD117 (55–80%), CD43 (9–100%), and Ki-67 (50–95%) [1, 17, 18] were consistent with the results of this study. To further subclassify patients after diagnosis of iMS or synchronous intramedullary AML, baseline evaluation using CT/MRI, PET/CT, bone marrow biopsy, and MICM typing is recommended.

Previous studies have shown that, without intervention, the median time of progression from MS to AML is 10 months [1]. Therefore, for gynecological oncologists, the time window between the first symptoms and treatment requires is particularly important. In the present study, the median interval between the first symptoms and initial chemotherapy was 3.5 months (range: 2–9); the interval was 2 months, for the two patients with bone marrow involvement of AML-M2. In addition, Patient 2 underwent three surgeries prior to receiving chemotherapy, one for iMS in the uterus and two for recurrences in the vulva and breast, and it then took her another 8 months to receive the final radical chemotherapy for iMS; she then reached complete response without recurrence. Thus, the radical treatment for iMS in the female genital tract should be surgery and postoperative chemotherapy. The extent of resection should be at the surgeon’s discretion and less than that of radical operations for cervical, endometrial, or ovarian cancer. Laparoscopic hysterectomy and bilateral salpingectomy are recommended for patients with isolated MS in cervix and uterus. For patients with isolated MS in pelvis and ovary, pelvic mass resection or ovary resection are recommended. The chemotherapy regimen like daunorubicin and cytarabine is recommended as the standard regimen of MS. The enhanced recovery after surgery methods could be helpful for these patients to speed initiation of chemotherapy [19]. Two AML-M2 patients received direct chemotherapy without surgery. This suggests that upfront chemotherapy followed by surgery can be beneficial in patients with MS [1]. Lontos et al. [20] found no significant difference in the OS of iMS patients between those with localized treatment plus upfront chemotherapy and those with localized treatment alone. For patients with a heavy tumor burden or in poor health, urgent chemotherapy or radiotherapy might be the preferred option [10, 21, 22]. For refractory/relapsed patients, the treatments should include hematopoietic stem cell transplantation and targeted therapy, such as with IDH and FLT-3 inhibitors [23–25]. Therefore, the treatment of patients with iMS is similar to treatment for gynecological cancer such as uterus sarcoma [26], and even though the origin of uterine iMS) and uterus sarcoma are different, patients could receive similar benefits from operation and postoperative chemotherapy.

The prognosis of patients with MS depends mainly on the MICM type, in which the karyotype and gene mutations are important, and on treatment. Begna et al. [27] found that patients with iMS had a better prognosis than those with MS with synchronous intramedullary AML (median OS: 78 vs. 16 months). A retrospective analysis of 56 patients with iMS, in which skin masses (34%) were the most frequent tumors, demonstrated that 75% of patients achieved response after frontline intensive chemotherapy, with a median OS of 3.41 years. In addition, the inv(16) translocation and mutations in the RAS pathway, DNMT3A, NPM1, IDH2, JAK2, KRAS, PTPN11, TET2, BCOR, and RAD21 have been found in MS patients [28]. In our study, MLL/AF9 gene fusions, and CEBPA, JAK2, NRAS, and FLT3-TKD mutations were also found. In the present study, the unfavorable prognostic factors included a poor response to chemotherapy, short interval to leukemia occurrence, heavy tumor burden (> 10 cm), and higher single-nucleotide variant number [29].

Although MS is rare in patients at gynecology clinics, gynecological oncologists should be familiar with the diagnosis and treatment of patients with isolated MS. Operation and chemotherapy are the main tools in the treatment of these patients, and timely referral to the department of hematology is helpful for patients to get MICM features. Future research with larger sample size is warranted to compare the oncological outcomes of patients receiving surgery and chemotherapy and chemotherapy alone.

The strength of this study is that it is a case series study providing real-world evidence of diagnosis and treatment in patients with iMS found in department of gynecological oncology. This study also has some limitations. First, retrospective case series are associated with the risk of selection bias. Second, case series do not have a control group, limiting conclusions about treatment efficacy. Finally, the sample size was small due to the rarity of this disease.

In conclusion, MS that mimics gynecological tumor is rare, and diagnosis should be confirmed by biopsy or postoperative pathology. For patients with iMS, chemotherapy and surgery are the radical treatment procedure, but initial chemotherapy alone should be considered first for patients with intramedullary AML. Poor response to chemotherapy, short interval to leukemia occurrence, and heavy tumor burden (> 10 cm) are considered unfavorable prognostic factors for patients with MS.

## Data Availability

The datasets used and/or analysed during the current study are available from the corresponding author on reasonable request.

## Abbreviations

MS: Myeloid sarcoma
AML: Acute myeloid leukemia
iMS: Isolated MS
OS: Overall survival
MICM: Morphology, Immunology, Cytogenetics, and Molecular biology
